# Carbohydrate-active enzyme architectures reflect distinct fungal and bacterial strategies for lignocellulose conversion to volatile fatty acids

**DOI:** 10.1093/ismeco/ycag262

**Published:** 2026-09-11

**Authors:** Christopher E Lawson, Joel P Howard, Thomas S Lankiewicz, James B Brown, Steven W Singer, Héctor García Martín, Michelle A O’Malley

**Affiliations:** Joint BioEnergy Institute, 5885 Hollis St 4th Floor, Emeryville, CA 94608, United States; Biological Systems and Engineering Division, Lawrence Berkeley National Laboratory, 1 Cyclotron Rd, Berkeley, CA 94720, United States; Department of Chemical Engineering and Applied Chemistry, University of Toronto, 200 College Street, Toronto, ON M5S 3E5, Canada; Department of Chemical Engineering and Applied Chemistry, University of Toronto, 200 College Street, Toronto, ON M5S 3E5, Canada; Joint BioEnergy Institute, 5885 Hollis St 4th Floor, Emeryville, CA 94608, United States; Department of Chemical Engineering, University of California, Santa Barbara, 3357 Engrg II UCSB, Santa Barbara, CA 93106, United States; Biological Systems and Engineering Division, Lawrence Berkeley National Laboratory, 1 Cyclotron Rd, Berkeley, CA 94720, United States; Joint BioEnergy Institute, 5885 Hollis St 4th Floor, Emeryville, CA 94608, United States; Biological Systems and Engineering Division, Lawrence Berkeley National Laboratory, 1 Cyclotron Rd, Berkeley, CA 94720, United States; Joint BioEnergy Institute, 5885 Hollis St 4th Floor, Emeryville, CA 94608, United States; Biological Systems and Engineering Division, Lawrence Berkeley National Laboratory, 1 Cyclotron Rd, Berkeley, CA 94720, United States; DOE Agile BioFoundry, Emery Station East, 5885 Hollis Street, Emeryville, CA 94608, United States; BCAM, Basque Center for Applied Mathematics, Mazarredo Zumarkalea, 14, Abando, 48009 Bilbao, Bizkaia, Spain; Joint BioEnergy Institute, 5885 Hollis St 4th Floor, Emeryville, CA 94608, United States; Department of Chemical Engineering, University of California, Santa Barbara, 3357 Engrg II UCSB, Santa Barbara, CA 93106, United States; Department of Bioengineering, University of California, Santa Barbara, Bioengineering Building, Room 1101494 UCEN Rd, Santa Barbara, CA 93106-5170, United States

**Keywords:** anaerobic fungi, anaerobic bacteria, carbohydrate active enzymes, metatranscriptomics, volatile fatty acids, lignocellulose, industrial biomanufacturing

## Abstract

Anaerobic fermentation with microbial communities is an emerging platform for converting lignocellulosic biomass to biofuels and bioproducts. The process relies on diverse anaerobic microbes that interact to deconstruct and convert lignocellulosic biomass into a range of products, such as volatile fatty acids (VFAs), through arresting methanogenesis during fermentation. However, defining the distinct functional roles played by various fungi and bacteria during anaerobic biodegradation remains poorly understood. Here, we performed parallel enrichment experiments from cow feces, goat feces, and anaerobic digester sludge, selecting for fungal- or bacterial-dominated communities that convert sorghum biomass into VFAs. Subsequently, we reconstructed metabolic networks across these enrichments using recovered bacterial metagenome-assembled genomes (MAGs), fungal isolate genomes, and genome-centric metatranscriptomics. This enabled direct comparison of the carbohydrate-active enzymes and fermentation pathways expressed by bacterial and fungal communities during lignocellulose conversion to VFAs. Our findings implicate diverse bacteria affiliated with the Bacteroidales and Lachnospiraceae in the direct conversion of lignocellulosic biomass to propionate and butyrate, respectively, whereas *Neocallimastix*-dominated fungal enrichments converted lignocellulose to lactate, acetate, and formate. Analysis of carbohydrate-active enzymes revealed that bacterial and fungal communities primarily targeted the same dominant lignocellulosic substrates while employing distinct enzyme repertoires and multidomain carbohydrate-active enzyme architectures, highlighting complementary enzymatic strategies for accessing and deconstructing lignocellulosic biomass. We anticipate that these findings will help inform efforts to develop synthetic consortia with tailored functionality for low-cost conversion of lignocellulosic biomass to fuels and bio-based chemicals.

## Introduction

Biomanufacturing of chemicals from renewable lignocellulosic biomass has the potential to offset society’s reliance on fossil fuels. One biomanufacturing strategy involves the use of anaerobic microbial communities to deconstruct and convert lignocellulosic biomass into biogas (methane and carbon dioxide) via anaerobic digestion [1]. While biogas can be upgraded to produce bioenergy (i.e. renewable natural gas or electricity), its market value is low, often rendering anaerobic digestion not economically viable. Therefore, higher value chemicals, such as volatile fatty acids (VFAs; $1–5/kg selling price), represent an emerging target product for anaerobic digestion that can be achieved by arresting methanogenesis during fermentation. These products can be chemically upgraded to liquid biofuels [2] or used directly in consumer products, antimicrobials, or industrial materials [3].

Top-down enrichment strategies are increasingly used to generate simplified, yet functionally stable, lignocellulose-degrading microbial communities while retaining key interactions present in natural systems [4, 5]. By repeatedly culturing complex inocula on a defined substrate under selective conditions, these enrichments reduce community complexity and enrich for organisms contributing to the desired function, providing tractable model systems for studying microbial ecology and metabolism. From an ecological perspective, these enrichment processes impose strong selection on community assembly, with substrate complexity and resource availability defining ecological niches that can promote niche partitioning, functional complementarity, and competition among community members [6]. Because both inoculum source and substrate are major determinants of microbial community assembly and function [4, 5], comparing enrichments derived from different environments provides an opportunity to distinguish conserved functional traits from source-specific adaptations. Furthermore, although anaerobic fungi and bacteria are well known to coexist during lignocellulose degradation in the rumen [7], the extent to which they partition biomass deconstruction through specialization on different plant cell wall components versus distinct enzymatic strategies remains poorly understood.

Several studies have demonstrated VFA production via arrested methanogenesis from different organic feedstocks [8]. Conversion of lignocellulosic biomass has largely been achieved by anaerobic fermentations using rumen contents as inocula, resulting in acetate, propionate, and butyrate (C2-C4 VFAs) as the main fermentation products in the absence of an external electron donor [9–11]. During fermentation, the microbiomes responsible for lignocellulose deconstruction and conversion have been reported as containing diverse ruminal bacteria [10, 11], with no studies reporting the presence of biomass degrading ruminal fungi, indicating that they are outcompeted by bacteria. While the overall metabolic processes mediated by ruminal bacteria during lignocellulose degradation to VFAs are generally understood, the specific metabolic networks and interactions driving *in situ* microbiome metabolism remain largely unresolved. Moreover, the contribution and specific metabolic activities expressed by ruminal fungi during lignocellulose deconstruction are only starting to emerge [12].

We hypothesized that bacterial- and fungal-dominated enrichments would retain broadly similar lignocellulose-degrading capabilities but employ distinct CAZyme repertoires and metabolic strategies that are influenced by inoculum source and the dominant members of the enrichment community. To test this hypothesis, we enriched five parallel anaerobic fungal and bacterial consortia derived from the cow and goat rumen and an anaerobic digester on sorghum substrate in chemically defined media selecting for VFA production. We quantified metabolite and gas production across the enrichments to examine functional differences between bacterial-dominated consortia and fungal-dominated consortia, as well as inoculum source. Subsequently, we reconstructed metabolic networks across the enrichments based on recovered bacterial metagenome-assembled genomes (MAGs) and fungal isolate genomes and profiled their gene expression using metatranscriptomics. Our results identify specific organisms, CAZymes, and metabolic pathways active in the deconstruction and conversion of sorghum biomass to VFAs. These results were used to infer key metabolic interactions responsible for producing specific VFA end products and activities that compete with VFA production. Together, these findings offer molecular-level insights on anaerobic microbiome function and will inform the rational design of microbiomes for improved lignocellulose conversion to valuable chemicals.

## Methods and materials

### Enrichment and cultivation of anaerobic bacterial and fungal cultures on sorghum biomass

Anaerobic consortia enriched for biomass-degrading bacteria and biomass-degrading fungi (herein termed “enrichments”) were cultivated in anaerobic Hungate tubes on 1% (w/w) sorghum (2 mm mill size; Idaho National Laboratory) as a sole substrate in 10 ml of chemically defined M2 media [13] with a ~85% N_2_, 12% CO_2_, and 3% H_2_ headspace and resazurin (0.1% w/v) added as a visual redox indicator. Cultures were initially inoculated inside an anaerobic chamber (AS-580, Anaerobe Systems) with 1 ml of either cow (5 g wet feces in 25 ml M2 media) or goat (3 pellets ~2.5 g in 25 ml M2) fecal solutions, or with 0.5 ml of sludge from an anaerobic digester to a final volume of 10 ml M2 media in the presence of either 5 mM chloramphenicol (CH, selecting for fungi) or 5 mM 2-bromoethanesulfonate (BES, selecting against methanogens) and passaged every 3 to 4 days based on 10% (v/v) transfers. BES was included to arrest methanogenesis and enrich for VFA-producing communities. Previous studies have shown no measurable direct effects of BES on non-methanogenic microorganisms at concentrations exceeding those used here, although inhibition of methanogenesis is expected to alter hydrogen turnover and potentially the activity of other hydrogen-utilizing microorganisms [14]. Accordingly, the bacterial enrichments represent lignocellulose-degrading communities under arrested methanogenesis and are not intended to reproduce native rumen fermentation. Enrichment cultures were maintained at a pH of 7–7.5 and a temperature of 39°C. This resulted in three bacterial enrichments from the cow, goat, and digester inocula (cow+BES, goat+BES, digester+BES) and two fungal enrichments from the cow and goat inocula (cow+CH, goat+CH). Cow and digester enrichments were passaged for 10 generations (~40 days), while goat enrichments were passaged for 5 generations (~20 days). Negative control cultures containing autoclaved sorghum in M2 media without inoculum were also included and showed no activity throughout the experiment. Analytical measurements were made on biological triplicates from each of the 5 enrichments cultures daily over a 4-day period. DNA/RNA samples were collected on Day 3 for metagenomic and metatranscriptomic analysis (see methods below).

### Measurement of fermentation products and total pressure accumulation

Total pressure accumulation in the headspace of Hungate tubes was monitored daily using a pressure transducer method as described previously [15]. Quantification of liquid fermentation products, including formate, acetate, propionate, butyrate, valerate, succinate, and lactate, was performed on a 1260 Infinity high-performance liquid chromatography (HPLC) system equipped with an Aminex HPX-87H column (part no. 1250140, Bio-Rad) with an inline 0.22 μm filter (Part No. 50671551, Agilent) followed by a Micro-Guard Cation H guard column (Part No. 1250129, Bio-Rad, Hercules, CA, USA) before the analytical column. Samples were prepared by acidification to a concentration of 5 mM H_2_SO_4_, followed by incubation at room temperature for 5 min, centrifugation at 21 000× g for 5 min, and filtration through a 0.22 μm polyethersulfone (PES) membrane filter into HPLC vials with 300 μl polypropylene inserts. Metabolites were separated using a mobile phase of 5 mM sulfuric acid at 50°C with a flow rate of 0.6 ml/min, a run length of 30 min, and an injection volume of 20 μl. Signals were measured using a variable wavelength detector (λ = 210 nm), and standard curves for each compound were made at 5 concentrations bracketing the range expected in the samples.

Quantification of hydrogen (H_2_) and methane (CH_4_) gas in the headspace of Hungate tubes were analyzed on a Thermo Fisher Scientific TRACE 1300 gas chromatograph (GC) equipped with a TracePLOT™ TG-BOND Msieve 5 A (Part No. 26003-6100, Thermo Fisher Scientific) and an Instant Connect Pulsed Discharge Detector (PDD) (Part No. 19070014, Thermo Fisher Scientific); 100 μL of headspace gas was collected and subsequently purged 3 times in a 100 μL air-tight syringe and needle. Then, 20 μL of headspace gas was collected and injected directly into the GC. The oven temperature for each run was 30°C, and the PDD temperature was 150°C. High-purity helium (Part No. HE 5.0UH-55, Praxair, Danbury, CT, USA) was further purified with a heated helium purifier (Part No. HP2, VICI) and used as the carrier gas with a flow rate of 0.5 ml/min. The same flushing and analysis procedures were followed for methane and hydrogen standards including 500 ppm H2, 2% H2, 5% H2, 20% H2, 0.5% CH4, 1% CH4, 5% CH4, 10% CH4, and 20% CH4 with balance helium (Douglas Fluid & Integration Technology, Prosperity, SC), which were run at each measurement timepoint to account for the PDD baseline that varied slightly each day.

### Extraction and sequencing of DNA and RNA

Biomass samples from bacterial enrichment cultures (cow, goat, digester) were collected for DNA and RNA extraction after 72 h (Day 3) following culture transfer. DNA was collected from bacterial enrichment samples in triplicate, while RNA was collected from bacterial and fungal enrichment samples in duplicate. Hungate tube contents were centrifuged at max speed for 5 min at 4°C, and biomass pellets were immediately flash frozen in liquid nitrogen. RNAlater was added to samples intended for RNA extraction prior to centrifugation. DNA was extracted from two replicates using the QIAGEN PowerSoil® DNA Isolation Kit (Qiagen, Hilden, Germany) and one replicate using a CTAB protocol. This was done to improve differential coverage binning, where only duplicate DNA samples from the PowerSoil® Kit extraction were used for assessing MAG relative abundance. RNA was extracted from duplicate samples using the QIAGEN RNeasy® Mini Kit with on-column DNAase 1 digestion (Qiagen, Hilden, Germany). DNA and RNA sequencing was performed on the Illumina MiSeq System (v2, 600 cycles) using the Nextera XT DNA Library Preparation Kit (Illumina, CA, USA) and the NEBNext® Ultra II Directional RNA Library Prep Kit at the Biological NanoStructures Lab at UC Santa Barbara. For bacterial metatranscriptomes, rRNA was depleted using the NEBNext® rRNA Depletion Kit (New England Biolabs, MA, USA). For fungal metatranscriptomes, enrichment of mRNA and separation from rRNA was achieved using the NEBNext® Poly(A) mRNA Magnetic Isolation Module. Strand-specific complementary DNA (cDNA) libraries were prepared with Invitrogen SuperScript II Reverse Transcriptase (Thermo Fisher Scientific, MA, USA). Nucleic acid quantity and quality were determined using a Qubit fluorometer (Thermo Fisher Scientific, MA, USA) and an Agilent TapeStation system (Agilent, CA, USA), respectively.

### Metagenomic assembly, binning, metabolic reconstruction

Raw paired-end DNA reads were quality-trimmed and filtered, adapter-trimmed, and contaminant-filtered using BBDuk v38.75, and read quality was assessed using FASTQC v0.11.8. Trimmed reads from the same sample (cow, goat, and digester bacterial enrichments) were co-assembled using MetaSPAades v3.15.3 [16] and mapped to assembled contigs using BBMap v38.75 with minid = 0.95. Resulting contigs of at least 1000 bp were binned with MaxBin2 v2.2.7 [17], MetaBAT2 v2.1512 [18], and CONCOCT v1.1.0 [19] and dereplicated using DAS-Tool v1.1.213 [20]. MAGs were taxonomically classified with GTDB-Tk v2.1.1 (release 9514) [21]. Bacterial MAGs were manually assigned a gram stain based on the taxonomic classification and literature references. Subsequently, gram stain-specific prediction of protein product localization for each open reading frame was performed using PSORTb v3.0 [22]. Estimates of MAG completeness and contamination was performed using CheckM v1.1.2 [23]. Bacterial MAGs were annotated using the NCBI Prokaryotic Genome Annotation Pipeline version 6.10 [24]. Metabolic pathways were annotated across MAGs using gapseq v1.4.0 using the parameters “gapseq find,” “all pathways,” and “bacterial mode” [25].

### Metatranscriptomic analysis

Complementary DNA reads were quality filtered as described above for DNA. SortMeRNA [26] was used to remove rRNA sequences using multiple databases for RNA sequences. The remaining non-rRNA reads were used for mapping. Fungal reference genomes available at the time of analysis, *Neocallimastix lanati* (Neolan1) [27], *Anaeromyces* sp. S4 (Anasp1) [28], *Orpinomyces* sp. strain C1A (Orpsp1) [28], *Piromyces* sp. E2 (PirE2) [28], *Piromyces sp.finn* (Pirfi3) [28], *Neocallimastix californiae G1* (Neosp1) [28], and *Neocallimastix frontalis var. giraffae* (NeoGfMa1) [29], were retrieved from the Joint Genome Institute MycoCosm database. An additional six fungal geomes *Piromyces* sp. UH3-1 (Piromy) [30], *Pecoramyces* sp. F1 (Pecora1) [31], *Neocallimastix* sp. WI3-B (NeoWI3), *Neocallimastix* sp. FC1 (NeoFC1) [32], *Neocallimastix constans* G3 (Neocon1) [33], and *Caecomyces churrovis* (Caecom1) [34] were collected, and RNA reads from fungal enrichment cultures were re-aligned to the combined 13 fungal reference genomes. The inclusion of the six additional genomes did not significantly change the number of reads mapped (<1%, data not shown), and therefore, they were not subject to further analysis. RNA reads from fungal enrichment cultures were aligned to a combined fungal reference genome (concatenation of all retrieved fungal reference genomes listed above) using the bbwrap.sh command from BBMap v38.86 with the usejni parameter set to true, ten threads and all other parameters set to their default values. Bacterial RNA reads were aligned competitively against the total metagenomic assembly using the parameter minid = 0.95 and then analyzed per MAG. Aligned reads were counted using the featureCounts command from Subread v2.0.3 using ten threads and all other parameters set to their default values. CAZymes and CAZyme motifs in fungal genomes and bacterial MAGs were predicted using dbcanLight v1.0.2 [35] using “cazyme mode” and “sub mode” options. If multiple distinct CAZyme motifs or substrate targets were predicted for the same open reading frame, predictions were concatenated into a single feature. Prediction of protein product localization for each CAZyme-encoding open reading frame was performed using SignalP 6.0 [36].

For fungal genomes and bacterial MAGs, dbCAN3 CAZyme motif and substrate target predictions, along with psortb (for bacteria) or SignalP (for fungi) localization predictions, were mapped to the read count data, generating a CAZyme expression matrix. Read counts from each sequencing sample were normalized for depth and gene length by calculating transcripts per million (TPM). For bacterial MAGs, TPM values assigned to the same CAZyme motif, in the same genome, from the same sample, and the same localization, were summed. The mean TPM values across replicate samples were calculated and used for downstream analysis. For each enrichment source (digester, cow, or goat), bacterial MAGs in the upper quantile of CAZyme expression, and CAZyme features predicted by PSORTb to be extracellular, were retained for differential expression analysis. Differential expression analysis was performed on bacterial MAGs using a Wilcoxon rank sum test (wilcoxoauc function from the presto R-package, v1.0.0). For fungal samples, TPM values assigned to the same CAZyme motif, from the same sample, and the same localization, were summed, and mean TPM values across replicate samples were calculated and used for downstream analysis.

## Results

### Anaerobic fungal and bacterial enrichments efficiently degrade lignocellulose to VFAs

To establish different fungal and bacterial consortia capable of degrading crude lignocellulose into VFAs, we first cultivated parallel anaerobic enrichment cultures on 1% milled sorghum in chemically defined M2 media (see Methods). Cultures were inoculated anaerobically with either cow or goat fecal pellets, or with sludge from an anaerobic digester in the presence of either chloramphenicol (selecting for fungi) or 2-bromoethanesulfonate (BES, selecting against methanogens) and passaged every 3–4 days over multiple generations (Fig. 1A). In total, inoculation of sorghum media with cow and goat feces produced stable fungal and bacterial enrichments from each source, whereas inoculation with anaerobic digester sludge only produced a bacterial enrichment, suggesting the source digester had low (or no) anaerobic fungal abundance.

**Figure 1:**
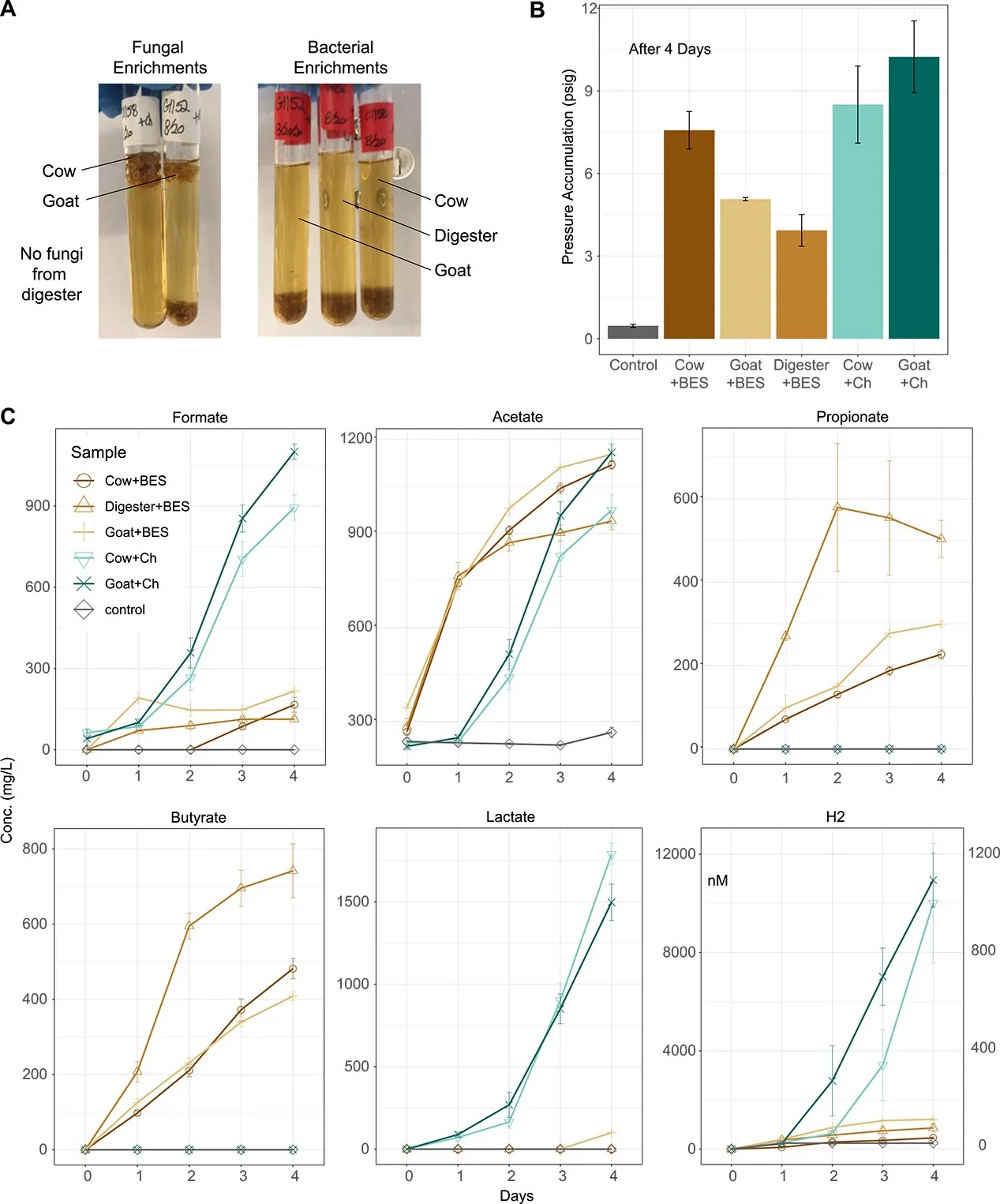
Fungal and bacterial enrichment cultures converting lignocellulosic biomass to VFAs. (A) Enrichment of anaerobic fungal and bacterial consortia from the cow and goat rumen and an anaerobic digester. (B) Accumulated gas production (psig) after 4 days and (C) VFA, lactate, and H_2_ production by anaerobic fungal and bacterial enrichments sourced from the cow and goat rumen and an anaerobic digester. VFA and lactate concentrations reported in mg/L, H_2_ concentration reported in nanomolar. H_2_ concentrations for fungal and bacterial enrichments shown on left and right *y*-axes, respectively.

All enrichments were capable of sorghum deconstruction based on large increases in pressure accumulation during 4-day Hungate tube (i.e. batch) fermentations (Fig. 1B). Fermentation products produced by the fungal enrichments were consistent with a bacterial-like mixed acid fermentation pathway previously reported for anaerobic fungi [37]. Both the cow and goat fungal enrichments produced high yields of formate, acetate, lactate, and hydrogen gas (Fig. 1C). In comparison, the three bacterial enrichments from the cow, goat, and digester inocula produced acetate, propionate, and butyrate (C2–C4 VFAs) as main fermentation products, as well as a small amount of hydrogen gas (Fig. 1C). These results are consistent with previous studies that quantified VFA production during lignocellulose degradation in anaerobic bacterial fermentations [9–11].

### Genome-centric metatranscriptomics reveals key microbes active across microbiomes

To identify key microbes responsible for lignocellulose degradation and conversion across the fungal and bacterial enrichments, we recovered genomes of individual species and profiled their gene expression using metatranscriptomics.

A total of 89 MAGs were recovered across the cow (33), goat (32), and digester (24) bacterial enrichments affiliated with diverse bacteria belonging to the phyla Actinomycetota, Bacillota, Bacteroidota, Desulfobacterota, Fusobacteriota, Pseudomonadota, Spirochaetota, and Synergistota. A summary of the genome statistics and taxonomy of these MAGs can be found in Supplementary Data 1. Mapping of DNA and RNA reads back to these genomes revealed that MAGs affiliated with Clostridia and Bacteroidetes dominated in abundance and activity across all bacterial enrichments (Fig. 2A–C). In particular, several MAGs seemed to be especially important to microbiome function based on their relative DNA and RNA abundance, including Lachnospiraceae-1C (25% DNA; 36% RNA), Prevotella-1C (13% DNA; 8% RNA), and Anaeroplasma-1C (3% DNA; 8% RNA) from the cow enrichment (Fig. 2B); Ruminococcaceae-1G (7% DNA; 19% RNA), Lachnospiraceae-4G (13% DNA; 19% RNA), Lachnospiraceae-5G (6% DNA; 10% RNA), Lachnospiraceae-7G (9% DNA; 5%RNA), Bacteroidales-1G (6% DNA; 12% RNA), and *Desulfovibrio*-2G (1% DNA; 6% RNA) from the goat enrichment (Fig. 2C); and *Bacteroides*-3D (31% DNA; 32% RNA), *Bacteroides*-1D (31% DNA; 7% RNA), and *Clostridium*-1D (12% DNA, 39% RNA) from the digester enrichment (Fig. 2A). Interestingly, several of the *Bacteroides* MAGs across the enrichments (e.g. *Bacteroides*-1C, *Bacteroides*-3G, and *Bacteroides*-2G) had very low relative RNA abundance compared to their relative DNA abundance. This likely reflects temporal differences in activity across the 4-day batch fermentation as RNA was collected on Day 3, suggesting these species were more active during the early stages of lignocellulose breakdown.

**Figure 2:**
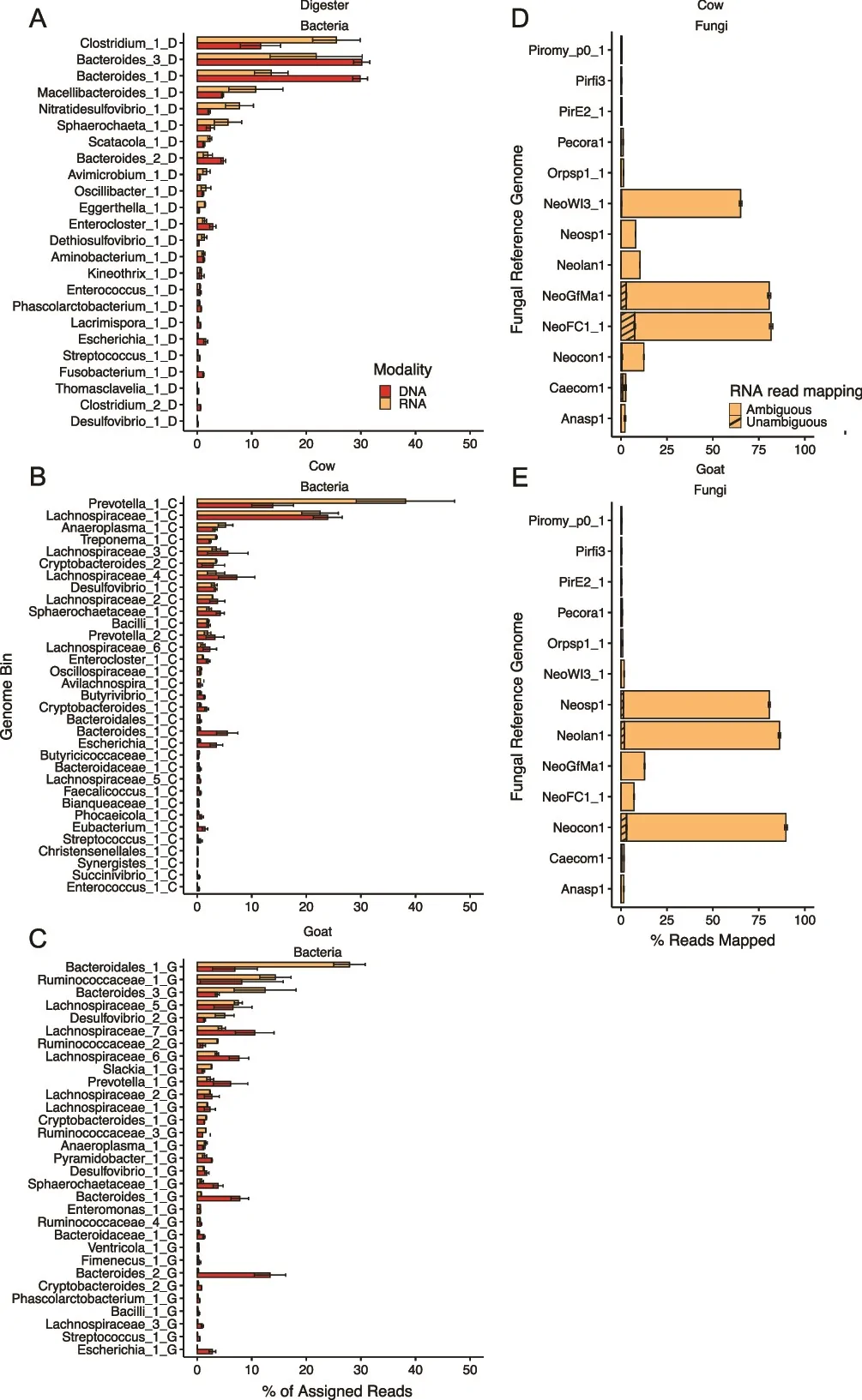
Abundance and activity of bacterial and fungal enrichments. The proportion of metagenomic (DNA) and metatranscriptomic (RNA) reads mapping to recovered MAGs from (A) digester bacterial enrichment, (B) cow bacterial enrichment, and (C) goat bacterial enrichment cultures. The percentage transcriptomic reads from (D) cow and (E) goat fungal enrichment cultures that mapped unambiguously and ambiguously to anaerobic fungal reference genomes. Bars represent the mean TPM and error bars represent ± standard deviation across *n* = 3 replicate DNA samples and *n* = 2 replicate RNA samples for each enrichment source. Fungal isolate genomes labels: Anasp1 - *A. robustus*, Neosp - *N. californiae G1*, NeoGfMa1 - *N. frontalis var. giraffae*, Neolan1 - *N. lanati*, Orpsp1 - *Orpinomyces sp*., PirE2 - Piromyces sp. E2, Pirfi3 - *P. finnis*, Piromy - Piromyces sp. UH3-1, Pecora1 - Pecoramyces sp. F1, NeoWI3 - *Neocallimastix* sp. WI3-B, NeoFC1 - *Neocallimastix* sp. FC1, Neocon1 - *N. constans* G3, and Caecom1 - *C. churrovis*.

To quantify gene expression in the fungal enrichments, RNA reads were mapped back to seven anaerobic fungi genomes available at the time of analysis, including *Anaeromyces robustus* (Anasp1), *N. californiae* G1 (Neosp1), *N. frontalis var. giraffae* (NeoGfMa1), *N. lanati* (Neolan1), *Orpinomyces* sp. (Orpsp1), *Piromyces* sp. E2 (PirE2), and *Piromyces finnis* (Pirfi3). A large fraction of RNA reads mapped successfully to these fungal references (~98%), confirming substantial transcriptional activity from anaerobic fungi, and that the collection of reference genomes sufficiently represented fungi in both enrichments (Fig. 2D; Supplementary Data 1). However, due to extensive sequence conservation and gene family expansion among anaerobic fungal genomes, many reads mapped ambiguously across closely related taxa (Fig. 2D), limiting our ability to confidently assign reads to specific species or genomes. Consequently, downstream analyses focused on functional profiles, especially CAZy family expression, rather than species-resolved transcript abundances. At a higher taxonomic level, read mapping patterns were broadly consistent with transcriptional activity from *Neocallimastix*-dominated enrichments (Fig. 2D), nevertheless high sequence similarity among genomes precluded robust species-level resolution.

### Carbohydrate active enzymes expression profiles indicate substrate specialization and niche partitioning during lignocellulose degradation

Sorghum biomass is predicted to consist of crystalline and amorphous cellulose that interacts with xylan containing frequent and irregular arabinosyl substitutions. The monosaccharide composition of non-cellulosic sorghum components in mole percent is ~63% xylose, 17% glucose, 12% arabinose, 4% galactose, 3% galacturonic acid, and 1% glucuronic acid [38]. This lignocellulosic matrix represents the primary carbon and energy substrates available to the enrichment cultures and was expected to drive microbiome structure and function [26]. To link the observed lignocellulose degradation activities to potential enzyme functions, we analyzed the expression of carbohydrate active enzymes (CAZymes) across all bacterial and fungal samples. This revealed a wide range of expressed CAZymes, targeting diverse glycosidic linkages present in cellulose, hemicellulose, starch, and pectin, especially cellulase (GH9), cellulose 1,4-β-cellobiosidase (GH48/GH6), and β-glucosidase (GH3) involved in cellulose degradation and endo-1,4-β-xylanase (GH10/GH11), α-N-arabinofuranosidase (GH43), xylan 1,4-β-xylosidase (GH43), and α -L-arabinofuranosidase (GH51) involved in hemicellulose degradation (Supplementary Data 2) that have previously been implicated in lignocellulose breakdown in the cow rumen [39].

Across the rumen (cow and goat) and digester enrichments, bacterial MAGs affiliated with Clostridia (Lachnospiraceae, *Ruminococcus*) and Bacteroidia (*Bacteroides*, *Macellibacteroides*, *Prevotella*) were the main contributors to CAZyme expression (Fig. 3; Supplementary Figure 1), consistent with their high abundance and activity based on read mapping (Fig. 2B and C). In the digester bacterial enrichment, *Clostridium*-1D was implicated as a key lignocellulose degrader with versatile hydrolytic activities based on expression of extracellular CAZymes involved in cellulose hydrolysis (GH9), xylan backbone cleavage (GH10/GH11), arabinoxylan debranching (GH43/GH51), and ester bond hydrolysis between ferulic acid and arabinose moieties in arabinoxylan (CE1) (Fig. 3A, Supplementary Data 2). As all these enzymes were extracellular, byproducts of degradation (xylo-oligosaccharides, sugar monomers, etc.) likely became available to other microbiome members.

**Figure 3:**
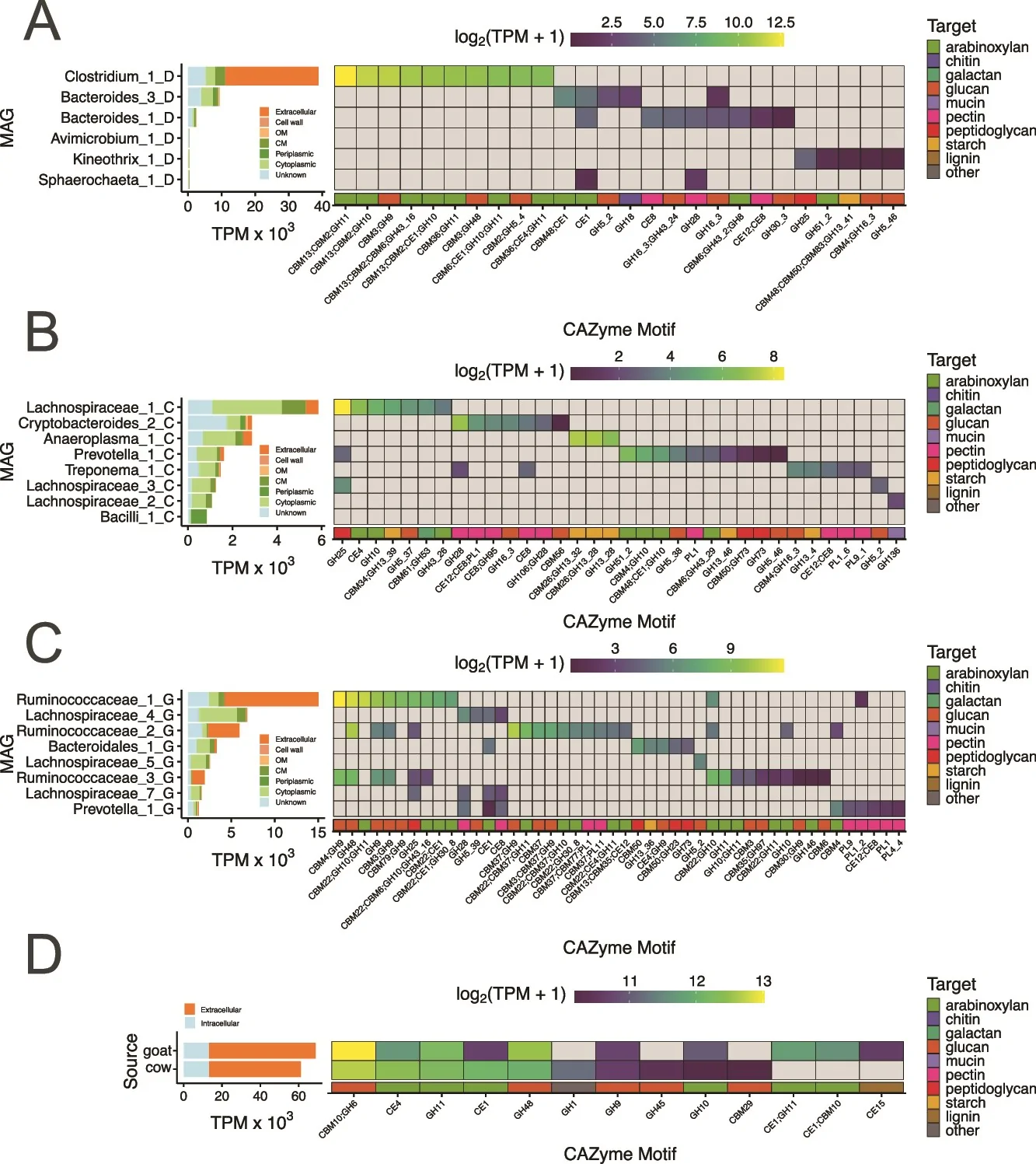
CAZyme localization and extracellular CAZyme expression profiles across bacterial and fungal enrichments. Left bar plots show the total expression of CAZymes and the PSORTb predicted localization for MAGs from (A) digester, (B) cow, and (C) goat bacterial enrichments in the upper quantile of gene expression. The corresponding heatmaps show the expression of up to the top 10 most differentially expressed extracellular CAZyme motifs (Wilcoxon rank sum test) between MAGs from the same enrichment. Colored tiles under the heatmaps indicate the predicted substrate target of the CAZyme motif based on dbCAN3 analysis and manual curation. (D) Left bar plots show the total expression of CAZymes and the SignalP predicted localization for cow and goat fungal enrichments. The corresponding heatmap shows the expression of the most expressed extracellular CAZyme motifs in the different enrichments.

In the goat bacterial enrichment, lignocellulose degradation activities appeared more distributed. Diverse *Ruminococcus* MAGs (*Ruminococcus*-1G*, Ruminococcus*-2G*, Ruminococcus*-3G) in the goat enrichment dominated CAZyme expression, including a wide array of CAZymes involved in cellulose and arabinoxylan hydrolysis (Fig. 3C, Supplementary Data 2). The three *Ruminococcus* MAGs expressed similar glycosyl hydrolase families, including GH9/GH48 and GH10/GH11, but differed in their associated carbohydrate-binding modules. For example, although all three MAGs expressed GH9 cellulases, *Ruminococcus*-1G and *Ruminococcus*-3G encoded GH9 linked to CBM4, whereas *Ruminococcus*-2G encoded GH9 linked to CBM37, indicating distinct multidomain architectures that may influence substrate binding.

Lignocellulose degradation activities were expressed across an even broader set of taxa in the cow bacterial enrichment, including Lachnospiraceae*-*1C, *Prevotella-*1C, and Bacteroidales-2C. Lachnospiraceae-1C and *Prevotella*-1C appeared to be the primary degraders of arabinoxylan, while Cryptobacteroides-2C and *Anaeroplasma*-1C targeted pectin and starch, respectively (Fig. 3B, Supplementary Data 2). Lachnospiraceae-1C had the highest expression of total and extracellular CAZymes and showed high expression of extracellular GH25-containing proteins together with diverse arabinoxylan-targeting enzymes. Although Lachnospiraceae-1C and *Prevotella*-1C both expressed GH10-family CAZymes, their multidomain architectures differed—GH10 proteins expressed by *Prevotella*-1C were linked to carbohydrate-binding modules (CBM4 and CBM48) and a carbohydrate esterase (CE1), whereas these domains were not associated with the GH10 proteins expressed by Lachnospiraceae-1C. These differences suggest that taxa targeting the same major polysaccharide may employ distinct substrate-binding and accessory enzyme strategies.

Across bacterial enrichments, several MAGs displayed high expression of multifunctional extracellular enzymes consisting of several glycosyl hydrolases, glycosyl hydrolases with carbohydrate esterases, or glycosyl hydrolyses with carbohydrate binding modules (Fig. 3, Supplementary Data 2; Supplementary Figure 1). For example, *Clostridium*-1D had high expression of a CAZyme encoding two endo-β-1,4-xylanases (GH10/GH11) with a carbohydrate esterase (CE1) that may improve hydrolysis of feruloylated arabinoxylan or oligosaccharides, whereas *Ruminococcus-*1G expressed CAZymes encoding Endo-β-1,4-xylanase (GH10) with a α-L-arabinofuranosidase (GH43), as well as a glucuronoarabinoxylan endo-1,4-beta-xylanase (GH30) with a carbohydrate esterase (CE1) that also likely target branched arabinoxylans (xylan with arabinosyl and feruloyl substitutions). These multifunctional enzymes were MAG specific, especially when comparing gene expression across abundant Lachnospiraceae MAGs recovered from the different enrichments that encoded widely varying lignocellulose hydrolysis capabilities, with *Clostridium*-1D appearing to be the most robust degrader. Taken together, these results highlight distinct CAZyme repertoires and multidomain architectures among bacterial enrichments derived from different inoculum sources, demonstrating multiple enzymatic solutions for lignocellulose deconstruction.

Anaerobic fungal enrichments from the cow and goat samples also had high expression of diverse CAZymes. The repertoire of expressed CAZymes in the goat and cow enrichments were highly similar and primarily targeted arabinoxylan and glucan fractions of the lignocellulosic biomass (Fig. 3D). This suggested that, although different fungi dominated, the cow and goat fungal enrichments performed similar biochemical functions shaped by sorghum as a substrate. Compared to bacterial enrichments from the same source, the fungal enrichments consistently expressed a higher diversity of extracellular CAZyme families, while the bacterial enrichments consistently expressed a higher diversity of non-extracellular CAZyme families, especially glycosyl hydrolases (GHs) (Fig. 4; Supplementary Figure 2). Comparing the extracellular fraction of fungal and bacterial CAZymes, the fungal enrichments expressed 37 and 30 GH-containing motifs from cow and goat enrichments, respectively, not expressed by in the bacterial enrichments, including the exo-type cellobiohydrolase, GH6 (Fig. 4; Supplementary Data 3). Conversely, the bacterial enrichments expressed 22 and 19 GH-containing motifs from cow and goat enrichments, respectively, not expressed by the fungal enrichments, such GH51_2, an α-L-arabinofuranosidase involved in hemicellulose degradation (Fig. 4; Supplementary Data 3).

**Figure 4:**
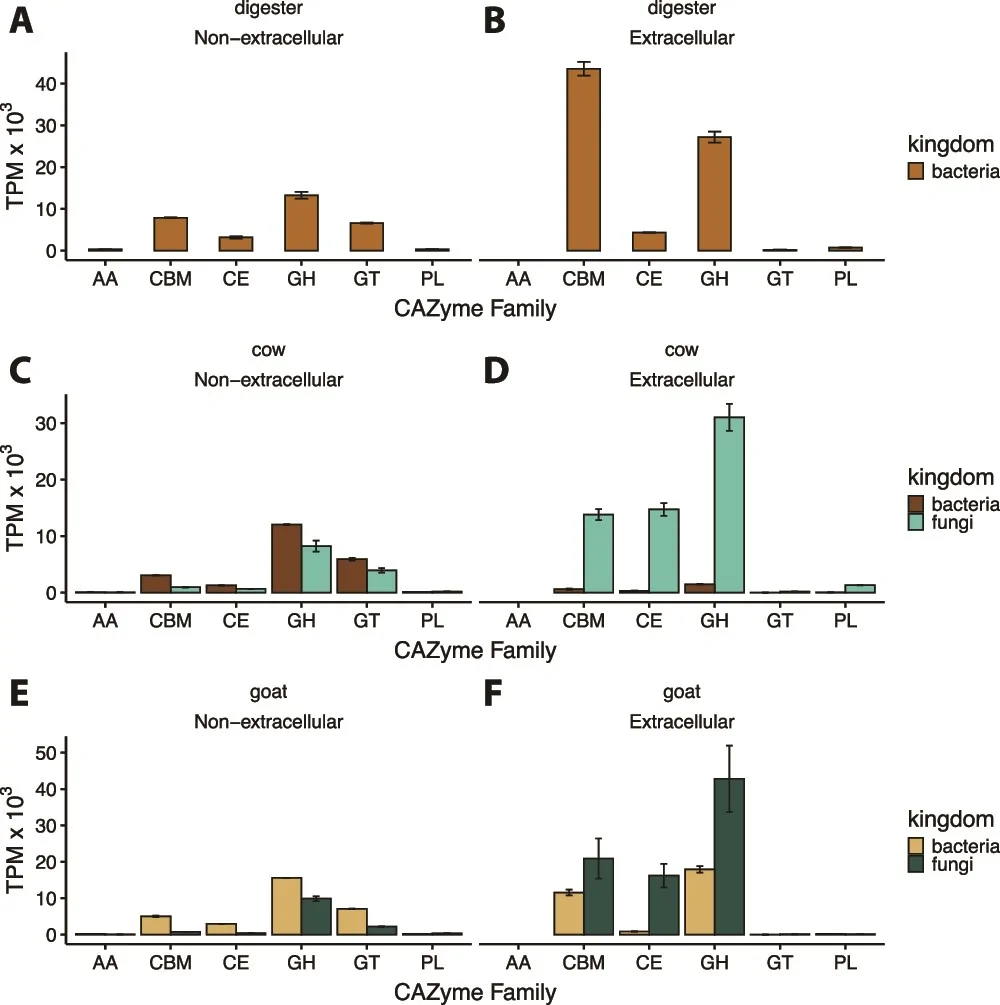
Expression and localization of CAZyme families across enrichment sources. Bar plots show the expression of CAZyme families—auxiliary activity (AA), carbohydrate-binding module (CBM), carbohydrate esterase (CE), glycoside hydrolase (GH), glycosyltransferase (GT), and polysaccharide lyase (PL)—expressed in digester (A, B), cow (C, D), and goat (E, F) enrichments. For each enrichment source, cazyme family expression is further partitioned into non-extracellular (A, C, E) and extracellular (B, D, F) fractions based on predicted subcellular localization using psotb or SignalP for bacterial or fungal enrichements, respectively. Bars represent the mean transcripts per million, and error bars represent ± standard deviation across *n* = 2 samples.

Rather than partitioning major plant cell-wall polysaccharides, fungal and bacterial enrichments primarily expressed CAZymes targeting the same dominant sorghum substrates, particularly glucan and arabinoxylan. However, they employed distinct enzyme repertoires and multidomain CAZyme architectures (Supplementary Figure 1). For example, anaerobic fungi showed high expression of the cellulase CBM10-GH6 together with CE4 carbohydrate esterases (Fig. 3D), whereas *Clostridium*-1D showed high expression of CBM13-CBM2-GH11/GH10 xylanases and CBM3-GH9 cellulases (Fig. 3A). Similarly, *Ruminococcaceae* MAGs expressed alternative multidomain architectures, including CBM4-GH9 and CBM22-GH10-GH11 (Fig. 3C). These observations suggest that anaerobic fungi and bacteria possess broadly overlapping lignocellulose-degrading capabilities while expressing distinct CAZyme repertoires and multidomain architectures. Some of these differences likely reflect lineage-specific evolution of CAZymes, whereas differences in carbohydrate-binding modules, multidomain architectures, and accessory activities may influence how these communities access and deconstruct the same lignocellulosic substrates.

Analysis of predicted protein localization further showed that CAZymes expression was dominated by extracellular enzymes for the digester bacterial enrichments and fungal (cow and goat) enrichments (Fig. 4), consistent with their primary role in lignocellulose depolymerization. Interestingly, this trend was less pronounced for the cow and goat bacterial enrichments, which had considerable expression of intracellular CAZymes predicted to target soluble carbohydrates, such as starch, sucrose, and beta-galactan, and xylan-derived oligosaccharides (Fig. 4C–F; Supplementary Data 2). This pattern is consistent with a transition from extracellular depolymerization of lignocellulose to intracellular metabolism of soluble carbohydrates during batch fermentation, although earlier extracellular degradation activities may have been underrepresented because transcriptomic profiling was performed only on Day 3.

Across all bacterial and fungal enrichments, genes encoding canonical lignin-depolymerizing enzymes, including lignin peroxidases, manganese peroxidases, and β-etherases, were not detected. Instead, transcripts encoding carbohydrate esterases (CE1) were detected across several abundant bacterial MAGs and anaerobic fungal genomes (Fig. 3, Supplementary Data 2 and 3), consistent with the potential to hydrolyze feruloyl- and *p*-coumaroyl ester linkages associated with sorghum arabinoxylan. Canonical pathways for the subsequent catabolism of released ferulic and/or *p*-coumaric acids (e.g. feruloyl-CoA synthetase, vanillate O-demethylase, or benzoyl-CoA degradation pathways) were not detected. However, phenylacetate-CoA ligase genes were across multiple bacterial MAGs, and near-complete phenylacetate catabolic pathways were detected in a small number of low-abundance MAGs (Enterobacteriaceae-1D, *Shigella*-1C; Supplementary Data 4). Together, these results suggest that lignin was not extensively depolymerized under the enrichment conditions, but was instead functionally circumvented through cleavage of lignin-carbohydrate complexes. However, because lignin-derived metabolites and structural changes to lignin were not directly measured, limited lignin depolymerization cannot be excluded. Importantly, this analysis does not preclude lignin modification via non-canonical or uncharacterized anaerobic mechanisms, as previously demonstrated by direct structural analyses of lignin during anaerobic fungal growth [28].

### Metatranscriptomic analysis reveals key fermentation pathways and microbes involved in VFA production from lignocellulosic sugars

To examine the metabolic networks of different fungal and bacterial consortia degrading lignocellulose into VFAs, we reconstructed anaerobic fermentation and respiration pathways across the recovered genomes and analyzed their gene expression. MAGs were assigned to different functional guilds based on gene expression of key enzymes and metabolic pathway responsible for specific substrate utilization and expected fermentation/respiration products. This analysis revealed five major guilds across the bacterial enrichments, including hydrolytic propionate-producing bacteria, hydrolytic butyrate-producing bacteria, hydrolytic alcohol-producing bacteria, succinate/lactate producing bacteria, and sulfate-reducing bacteria.

The most abundant and active MAGs were hydrolytic bacteria involved in complex polysaccharide fermentation to propionate or butyrate, together with acetate and H_2_ formation. Propionate production was dominated by MAGs affiliated with the order Bacteroidales, including *Prevotella*-1C and *Prevotella*-2C from the cow enrichment, *Bacteroides*-2G, Bacteroidales-1G, *Bacteroides*-1G, and *Prevotella*-1G from the goat enrichment, and *Bacteroides*-3D, *Bacteroides*-1D, and *Macellibacteroides*-1D from the digester enrichment (Fig. 5; Supplementary Dataset 4). These MAGs all expressed enzymes of the methylmalonyl-CoA pathway (Supplementary Dataset 4), including a sodium ion pumping methylmalonyl-CoA decarboxylase [40], that couples propionate formation with the generation of an ion motive force that can be used for ATP synthesis. These MAGs also had high expression of pathways for sugar utilization (glucose, xylose, and arabinose) (Fig. 5; Supplementary Dataset 4) consistent with their high expression of CAZymes involved in liberating these sugars during cellulose and hemicellulose degradation (Fig. 3).

**Figure 5:**
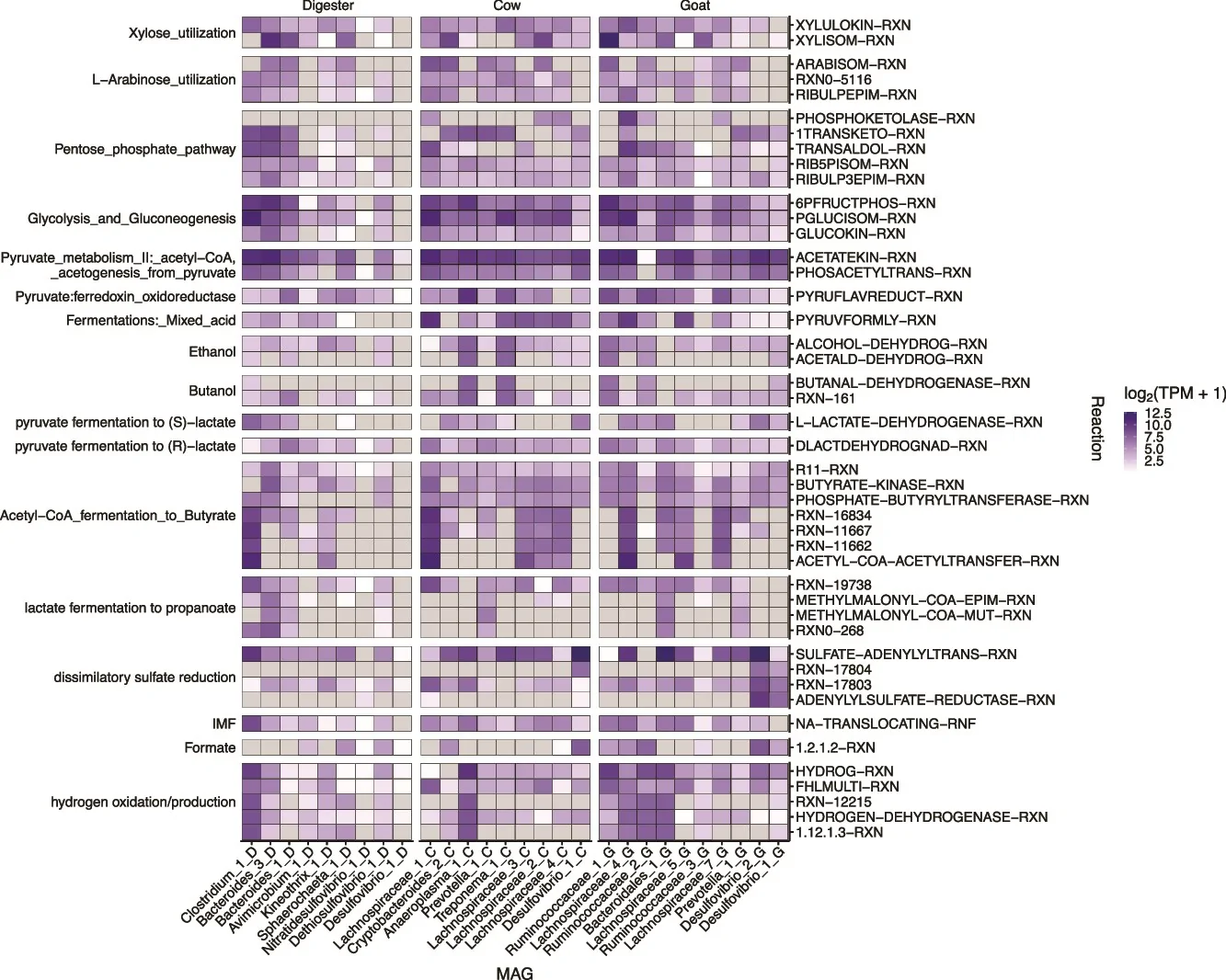
Expression of key metabolic pathways across MAGs from bacterial enrichments. Heatmap showing the expression of genes encoding key reactions in carbohydrate utilization and fermentation pathways, predicted from metabolic reconstructions (gapseq) across MAGs from digester (left), cow (middle), and goat (right) enrichments. Individual reactions within each pathway are listed on the right using MetaCyc reaction identifiers. Columns represent individual MAGs (labeled by taxon and enrichment source). Cell intensity indicates expression level, with empty cells denoting reactions not predicted to be encoded in that MAG.

Butyrate production across the enrichments was dominated by MAGs affiliated with the Lachnospiraceae (Fig. 5). All Lachnospiraceae MAGs had high gene expression of the reverse beta-oxidation pathway, including an electron bifurcating acyl-CoA dehydrogenase complex [29]. They also expressed the terminal enzymes phosphate butyryltransferase and butyrate kinase that couples butyrate synthesis with substrate level phosphorylation, except for *Clostridium*-1D, which instead expressed a butyryl-CoA:acetate CoA transferase [41]. While all Lachnospiraceae MAGs expressed diverse CAZymes (Fig. 3) and sugar utilization pathways (Fig. 5), *Clostridium*-1D from the digester enrichment especially stood out among the Lachnospiraceae MAGs as a specialized cellulose and hemicellulose degrader based on its high copy number and gene expression of 1,4-beta-xylanases, α-L-arabinofuranosidases, and cellulases (Fig. 3).

In addition to propionate and butyrate, several MAGs affiliated with the *Ruminococcus* and Sphaerochaetaceae were predicted to be involved in complex polysaccharide fermentation to ethanol, acetate, and H_2_ formation. *Ruminococcus*-1G and *Ruminococcus*-2G had high expression of a bifunctional acetaldehyde-CoA/alcohol dehydrogenase and NAD-dependent electron-bifurcating [FeFe]-hydrogenase (HydABC), together with CAZymes and substrate utilization pathways involved in both cellulose and hemicellulose degradation (Fig. 5; Supplementary Dataset 4). This metabolism is consistent with previous batch fermentation results from other *Ruminococcus* species, such as *Ruminococcus albus* isolated from the cow rumen [42, 43]. Diverse Sphaerochaetaceae MAGs, including *Treponema*-1C, Sphaerochaetaceae-1C, *Sphaerochaeta*-1D, and Sphaerochaetaceae-1C were also implicated in ethanol, acetate, formate, and H_2_ formation based on the high gene expression of alcohol dehydrogenases (iron-containing alcohol dehydrogenase and/or bifunctional acetaldehyde-CoA/alcohol dehydrogenase) and hydrogenases (iron-only hydrogenases and/or ferredoxin hydrogenases) (Fig. 5; Supplementary Dataset 4). Additionally, Sphaerochaetaceae-1G also had high expression of lactate dehydrogenase and hydroxyacid dehydrogenase suggesting they may also produce lactate as an end-product.

Aside from fermentation pathways, all enrichments contained bacteria affiliated with *Desulfovibrio sp.* predicted to be capable of anaerobic sulfate respiration to hydrogen sulfide coupled to ethanol, formate, lactate, and H_2_ oxidation. In particular, *Desulfovibrio*-1C, *Desulfovibrio*-1G, *Desulfovibrio*-2G, and *Nitratidesulfovibrio*-1D had high expression of sulfate adenylyltransferase, adenylylsulfate reductase, and dissimilatory sulfite reductase involved in dissimilatory sulfate respiration together with high expression of hydrogenases, formate dehydrogenase, lactate dehydrogenase, alcohol dehydrogenase, and aldehyde dehydrogenase (Fig. 5; Supplementary Dataset 4), suggesting co-utilization of ethanol, formate, lactate, and H_2_ for energy generation. This is consistent with the availability of these substrates as fermentation by-products (Fig. 1).

## Discussion

The use of genome-centric metatranscriptomics allowed us to analyze the metabolism and gene expression of five different anaerobic enrichment cultures degrading lignocellulosic biomass to VFAs. Our results reveal that across all bacterial enrichments, MAGs affiliated with the order Bacteroidales (*Bacteroides* and *Prevotella*) and family Lachnospiraceae were dominant lignocellulose degraders that fermented biomass-derived sugars to propionate and butyrate, respectively, together with acetate and H_2_ formation. Other MAGs affiliated with *Ruminococcus* from the goat bacterial enrichment, and to a lesser extent the family Sphaerochaetaceae across all bacterial enrichments, were also implicated in biomass breakdown to ethanol, lactate, formate, acetate, and H_2_ (*Ruminococcus,* Sphaerochaetaceae), consistent with the metabolism of closely related representative isolates [44, 45]. The resulting fermentation products, especially ethanol, formate, lactate, and H_2_, were predicted to be substrates for *Desulfovibrio* MAGs that performed dissimilatory sulfate reduction across all bacterial enrichments*.* The fungal enrichments degraded lignocellulose and employed a mixed acid fermentation pathway, producing lactate, acetate, ethanol, H_2_, and CO_2_ as major end products, consistent with the metabolism of representative isolates [27]. Taken together, these results highlight that substrate (sorghum biomass) and chemical inhibitors (chloramphenicol and BES) selected for microbiomes with similar overall functional guild structure, despite containing different taxa and starting from disparate inocula.

Detailed analysis of CAZyme gene expression revealed that bacterial and fungal communities shared broadly similar lignocellulose-degrading capabilities while employing distinct enzyme repertoires and multidomain carbohydrate-active enzyme architectures. While all bacterial and fungal enrichments expressed CAZymes necessary for cellulose and hemicellulose degradation, including those involved in cellulose hydrolysis (GH9), arabinoxylan backbone cleavage (GH10/GH11), and arabinoxylan debranching (GH43/GH51/CE1), differences in enzyme architectures and carbohydrate-binding modules based on multifunctional enzymes linking two or more CAZymes together with diverse CBMs were widespread. Many of these multifunctional enzymes frequently combined backbone-cleaving and accessory activities within individual multidomain proteins, such as xylan backbone cleavage with various debranching activities, likely conferring synergistic degradation effects as shown by purified enzyme studies [46]. The association of these enzymes with diverse CBMs also suggests targeted specialization towards specific lignocellulose structures (e.g. substituted backbones or branched linkages), consistent with recent observations among human gut microbiota [47]. These observations suggest that bacterial and fungal communities largely target the same dominant plant polysaccharides but employ distinct enzymatic strategies, including differences in carbohydrate-binding modules, multidomain enzyme architectures, and accessory activities, which may provide complementary mechanisms for biomass deconstruction.

Hydrolysis and subsequent fermentation of lignocellulosic substrates resulted in a range of fermentation acids shaped by culture conditions. Indeed, it has been shown that the spectrum of fermentation products is highly influenced by both substrate and environmental parameters, such as pH and temperature. In our study, a neutral pH of 7–7.5 and temperature of 39°C was maintained, selecting for bacterial enrichments that produced short-chain fatty acids (acetate, propionate, butyrate) and H_2_ as major fermentation products, and fungal enrichments that produced mixed acid fermentation products (lactate, acetate, formate) and H_2_. These acids appeared to be directly produced from lignocellulose-derived sugars versus indirectly via lactate as an intermediate, which can result via cross feeding between lactate-producing species (e.g. LAB, Bifidobacteria) and lactate-utilizing species. This may have resulted from having neutral versus mildly acid pH (~5–6) conditions, the latter of which has been shown to select for lactate cross-feeding. However, general principles on what controls the distribution of fermentation products remain unclear and represent an important knowledge gap to advance anaerobic fermentation as a platform for biorefining of specific volatile fatty acids, lactate, alcohols, and other bulk chemicals [48].

While further studies are still needed to comprehensively map the functional niches driving anaerobic lignocellulose degradation to VFAs, our results provide important insights into the complementary enzymatic strategies and metabolic interactions underpinning anaerobic lignocellulose degradation to VFAs. We anticipate that these findings will help inform efforts to develop synthetic microbiomes with tailored functionality for low-cost conversion of lignocellulosic biomass to fuels/chemicals [49]. This will require isolating and assembling novel anaerobes, such as those identified here, into defined consortia to eliminate competing metabolic pathways, as well as introducing novel functionalities via metabolic engineering [50]. Such consortia would also serve to elucidate basic principles governing microbiome assembly and function, resulting in robust anaerobic fermentation platforms for sustainable biomanufacturing.

## Supplementary Material

Supplementary_material_ycag262

## Data Availability

Raw DNA and cDNA read data and annotated metagenome assembled genomes (MAGs) can be found on the National Center for Biotechnology Information (NCBI) website under BioProject accession no. PRJNA1379681.

## References

[1] Sawatdeenarunat C, Surendra KC, Takara D. et al. Anaerobic digestion of lignocellulosic biomass: challenges and opportunities. *Bioresour Technol* 2015;178:178–86. 10.1016/j.biortech.2014.09.103 25446783

[2] Huq NA, Hafenstine GR, Huo X. et al. Toward net-zero sustainable aviation fuel with wet waste–derived volatile fatty acids, physical sciences. *Proc Natl Acad Sci* 2021;118. 10.1073/pnas.2023008118 PMC8020759 33723013

[3] Agnihotri S, Yin DM, Mahboubi A. et al. A glimpse of the world of volatile fatty acids production and application: a review. *Bioengineered* 2022;13:1249–75. 10.1080/21655979.2021.1996044 PMC8805862 34738864

[4] Peng X, Wilken SE, Lankiewicz TS. et al. Genomic and functional analyses of fungal and bacterial consortia that enable lignocellulose breakdown in goat gut microbiomes. *Nat Microbiol* 2021;6:499–511. 10.1038/s41564-020-00861-0 PMC8007473 33526884

[5] Wong MT, Wang W, Lacourt M. et al. Substrate-driven convergence of the microbial community in lignocellulose-amended enrichments of gut microflora from the Canadian beaver (Castor canadensis) and north American moose (Alces americanus). *Front Microbiol* 2016;7. 10.3389/fmicb.2016.00961 PMC4914502 27446004

[6] Jiménez DJ, Dini-Andreote F, DeAngelis KM. et al. Ecological insights into the dynamics of plant biomass-degrading microbial consortia. *Trends Microbiol* 2017;25:788–96. 10.1016/j.tim.2017.05.012 28648267

[7] Gruninger RJ, Puniya AK, Callaghan TM. et al. Anaerobic fungi (phylum Neocallimastigomycota): advances in understanding their taxonomy, life cycle, ecology, role and biotechnological potential. *FEMS Microbiol Ecol* 2014;90:1–17. 10.1111/1574-6941.12383 25046344

[8] Stamatopoulou P, Malkowski J, Conrado L. et al. Fermentation of organic residues to beneficial chemicals: a review of medium-chain fatty acid production. *Processes* 2020;8. 10.3390/pr8121571

[9] Weimer PJ, Nerdahl M, Brandl DJ. Production of medium-chain volatile fatty acids by mixed ruminal microorganisms is enhanced by ethanol in co-culture with Clostridium kluyveri. *Bioresour Technol* 2015;175:97–101. 10.1016/j.biortech.2014.10.054 25459809

[10] Agematu H, Takahashi T, Hamano Y. Continuous volatile fatty acid production from lignocellulosic biomass by a novel rumen-mimetic bioprocess. *J Biosci Bioeng* 2017;124:528–33. 10.1016/j.jbiosc.2017.06.006 28690158

[11] Fonoll X, Shrestha S, Khanal SK. et al. Understanding the anaerobic digestibility of lignocellulosic substrates using rumen content as a cosubstrate and an inoculum. *ACS EST Eng* 2021;1:424–35. 10.1021/acsestengg.0c00164

[12] Hagen LH, Brooke CG, Shaw CA. et al. Proteome specialization of anaerobic fungi during ruminal degradation of recalcitrant plant fiber. *ISME J* 2021;15:421–34. 10.1038/s41396-020-00769-x PMC8026616 32929206

[13] Teunissen MJ, Op den Camp HJM, Orpin CG. et al. Comparison of growth characteristics of anaerobic fungi isolated from ruminant and non-ruminant herbivores during cultivation in a defined medium. *Microbiology* 1991;137:1401–8. 10.1099/00221287-137-6-1401 1919514

[14] Ungerfeld EM . Metabolic hydrogen flows in rumen fermentation: principles and possibilities of interventions. *Front Microbiol* 2020;11. 10.3389/fmicb.2020.00589 PMC7174568 32351469

[15] Haitjema CH, Solomon KV, Henske JK. et al. Anaerobic gut fungi: advances in isolation, culture, and cellulolytic enzyme discovery for biofuel production. *Biotechnol Bioeng* 2014;111:1471–82. 10.1002/bit.25264 24788404

[16] Nurk S, Meleshko D, Korobeynikov A. et al. metaSPAdes: a new versatile metagenomic assembler. *Genome Res* 2017;27:824–34. 10.1101/gr.213959.116 PMC5411777 28298430

[17] Wu YW, Tang YH, Tringe SG. et al. MaxBin: an automated binning method to recover individual genomes from metagenomes using an expectation-maximization algorithm. *Microbiome* 2014;2:26. 10.1186/2049-2618-2-26 PMC4129434 25136443

[18] Kang DD, Froula J, Egan R. et al. MetaBAT, an efficient tool for accurately reconstructing single genomes from complex microbial communities. *PeerJ* 2015;3:e1165. 10.7717/peerj.1165 PMC4556158 26336640

[19] Alneberg J, Bjarnason BS, Bruijn I d. et al. Binning metagenomic contigs by coverage and composition. *Nat Methods* 2014;11:1144–6. 10.1038/nmeth.3103 25218180

[20] Sieber CMK, Probst AJ, Sharrar A. et al. Recovery of genomes from metagenomes via a dereplication, aggregation and scoring strategy. *Nat Microbiol* 2018;3:836–43. 10.1038/s41564-018-0171-1 PMC6786971 29807988

[21] Chaumeil PA, Mussig AJ, Hugenholtz P. et al. GTDB-Tk: a toolkit to classify genomes with the genome taxonomy database. *Bioinformatics* 2020;36:1925–7. 10.1093/bioinformatics/btz848 PMC7703759 31730192

[22] Yu NY, Wagner JR, Laird MR. et al. PSORTb 3.0: improved protein subcellular localization prediction with refined localization subcategories and predictive capabilities for all prokaryotes. *Bioinformatics* 2010;26:1608–15. 10.1093/bioinformatics/btq249 PMC2887053 20472543

[23] Parks DH, Imelfort M, Skennerton CT. et al. CheckM: assessing the quality of microbial genomes recovered from isolates, single cells, and metagenomes. *Genome Res* 2015;25:1043–55. 10.1101/gr.186072.114 PMC4484387 25977477

[24] Tatusova T, DiCuccio M, Badretdin A. et al. NCBI prokaryotic genome annotation pipeline. *Nucleic Acids Res* 2016;44:6614–24. 10.1093/nar/gkw569 PMC5001611 27342282

[25] Zimmermann J, Kaleta C, Waschina S. gapseq: informed prediction of bacterial metabolic pathways and reconstruction of accurate metabolic models. *Genome Biol* 2021;22:81. 10.1186/s13059-021-02295-1 PMC7949252 33691770

[26] Kopylova E, Noé L, Touzet H. SortMeRNA: fast and accurate filtering of ribosomal RNAs in metatranscriptomic data. *Bioinformatics* 2012;28:3211–7. 10.1093/bioinformatics/bts611 23071270

[27] Wilken SE, Monk JM, Leggieri PA. et al. Experimentally validated reconstruction and analysis of a genome-scale metabolic model of an anaerobic Neocallimastigomycota fungus. *mSystems* 2021;6:10.1128/msystems.00002-21. 10.1128/msystems.00002-21 PMC8561657 33594000

[28] Haitjema CH, Gilmore SP, Henske JK. et al. A parts list for fungal cellulosomes revealed by comparative genomics. *Nat Microbiol* 2017;2:1–8. 10.1038/nmicrobiol.2017.87 28555641

[29] Hooker CA, Hanafy R, Hillman ET. et al. A genetic engineering toolbox for the Lignocellulolytic anaerobic gut fungus Neocallimastix frontalis. *ACS Synth Biol* 2023;12:1034–45. 10.1021/acssynbio.2c00502 PMC11677189 36920337

[30] Hooker CA, Hillman ET, Overton JC. et al. Hydrolysis of untreated lignocellulosic feedstock is independent of S-lignin composition in newly classified anaerobic fungal isolate, Piromyces sp. UH3-1. *Biotechnol Biofuels* 2018;11:293. 10.1186/s13068-018-1292-8 PMC6203967 30386430

[31] Li Y, Li Y, Jin W. et al. Combined genomic, transcriptomic, proteomic, and physiological characterization of the growth of Pecoramyces sp. F1 in monoculture and co-culture with a syntrophic methanogen. *Front Microbiol* 2019;10. 10.3389/fmicb.2019.00435 PMC6414434 30894845

[32] Kim BCR, Blair EM, Howard JP. et al. Engineering anaerobic fungal-bacterial consortia for direct conversion of lignocellulosic biomass into medium-chain fatty acids. Preprint, bioRxiv, 3 Feb. 2026, 2025.08.19.670934. 10.1101/2025.08.19.670934

[33] Blair EM, Navaratna TA, Ahern CB. et al. Genomic and transcriptomic characterization of carbohydrate-active enzymes in the anaerobic fungus Neocallimastix cameroonii var. constans. *G3 (Bethesda)* 2025;15:jkaf137. 10.1093/g3journal/jkaf137 PMC12341934 40519056

[34] Brown JL, Swift CL, Mondo SJ. et al. Co-cultivation of the anaerobic fungus Caecomyces churrovis with Methanobacterium bryantii enhances transcription of carbohydrate binding modules, dockerins, and pyruvate formate lyases on specific substrates. Biotechnol Biofuels. 2021;14:234. 10.1186/s13068-021-02083-w PMC8665504 34893091

[35] Zheng J, Ge Q, Yan Y. et al. dbCAN3: automated carbohydrate-active enzyme and substrate annotation. *Nucleic Acids Res* 2023;51:W115–21. 10.1093/nar/gkad328 PMC10320055 37125649

[36] Teufel F, Almagro Armenteros JJ, Johansen AR. et al. SignalP 6.0 predicts all five types of signal peptides using protein language models. *Nat Biotechnol* 2022;40:1023–5. 10.1038/s41587-021-01156-3 PMC9287161 34980915

[37] Boxma B, Voncken F, Jannink S. et al. The Anaerobic Chytridiomycete Fungus Piromyces Sp. E2 Produces Ethanol via Pyruvate:Formate Lyase and an Alcohol Dehydrogenase E. Molecular Microbiology, 2004;51:1389–1399. 10.1046/j.1365-2958.2003.03912.x 14982632

[38] Gao Y, Lipton AS, Wittmer Y. et al. A grass-specific cellulose-xylan interaction dominates in sorghum secondary cell walls. Nat Commun 2020;11:6081. 10.1038/s41467-020-19837-z PMC7695714 33247125

[39] Comtet-Marre S, Parisot N, Lepercq P. et al. Metatranscriptomics reveals the active bacterial and eukaryotic fibrolytic communities in the rumen of dairy cow fed a mixed diet. *Front Microbiol* 2017;8:67. 10.3389/fmicb.2017.00067 PMC5281551 28197133

[40] Hilpert W, Dimroth P. Conversion of the chemical energy of methylmalonyl-CoA decarboxylation into a Na+gradient. *Nature* 1982;296:584–5. 10.1038/296584a0 7070502

[41] Louis P, Young P, Holtrop G. et al. Diversity of human colonic butyrate-producing bacteria revealed by analysis of the butyryl-CoA:acetate CoA-transferase gene. *Environ Microbiol* 2010;12: 304–14. 10.1111/j.1462-2920.2009.02066.x 19807780

[42] Zheng Y, Kahnt J, Kwon IH. et al. Hydrogen formation and its regulation in Ruminococcus albus: involvement of an electron-bifurcating [FeFe]-hydrogenase, of a non-electron-bifurcating [FeFe]-hydrogenase, and of a putative hydrogen-sensing [FeFe]-hydrogenase. *J Bacteriol* 2014;196:3840–52. 10.1128/jb.02070-14 PMC4248817 25157086

[43] Iannotti EL, Kafkewitz D, Wolin MJ. et al. Glucose fermentation products of Ruminococcus albus grown in continuous culture with vibrio succinogenes: changes caused by interspecies transfer of H2. *J Bacteriol* 1973;114:1231–40. 10.1128/jb.114.3.1231-1240.1973 PMC285387 4351387

[44] Paster BJ, Canale-Parola E. Treponema saccharophilum sp. nov., a large pectinolytic spirochete from the bovine rumen. *Appl Environ Microbiol* 1985;50:212–9. 10.1128/aem.50.2.212-219.1985 PMC238606 4051480

[45] Paster BJ, Canale-Parola E. Physiological diversity of rumen spirochetes. *Appl Environ Microbiol* 1982;43:686–93. 10.1128/aem.43.3.686-693.1982 PMC241895 7073277

[46] Sørensen HR, Jørgensen CT, Hansen CH. et al. A novel GH43 α-l-arabinofuranosidase from Humicola insolens: mode of action and synergy with GH51 α-l-arabinofuranosidases on wheat arabinoxylan. *Appl Microbiol Biotechnol* 2006;73:850–61. 10.1007/s00253-006-0543-y 16944135

[47] Yao T, Deemer DG, Chen MH. et al. Differences in fine arabinoxylan structures govern microbial selection and competition among human gut microbiota. *Carbohydr Polym* 2023;316:121039. 10.1016/j.carbpol.2023.121039 PMC13358863 37321733

[48] Regueira A, JuliusL R, Wahl SA. et al. Resource allocation explains lactic acid production in mixed-culture anaerobic fermentations. *Biotechnol Bioeng* 2021;118:745–58. 10.1002/bit.27605 33073364

[49] Lynd LR, Beckham GT, Guss AM. et al. Toward low-cost biological and hybrid biological/catalytic conversion of cellulosic biomass to fuels. *Energy Environ Sci* 2022;15:938–90. 10.1039/d1ee02540f

[50] Lawson CE . Retooling microbiome engineering for a sustainable future. *mSystems* 2021;6:10.1128/msystems.00925-21. 10.1128/msystems.00925-21 PMC12338127 34463582

