# Supplementary material for: Carbohydrate-active enzyme architectures reflect distinct fungal and bacterial strategies for lignocellulose conversion to volatile fatty acids
Source: ISME Commun. 2026 Sep 11;6(1):ycag262. doi: 10.1093/ismeco/ycag262 (PMC13641081; doi:10.1093/ismeco/ycag262)
Supplement: Supplementary file 1 [file supplementary_material_ycag262.zip › Supplementary Information.pdf]

SUPPLEMENTARY INFORMATION

Carbohydrate-active enzyme architectures reveal distinct enzymatic strategies of anaerobic fungi and bacteria during lignocellulose conversion to volatile fatty acids

Christopher E. Lawson[1,2,3,†], Joel P. Howard[3†], Thomas S. Lankiewicz[1,4], James B. Brown[2], Steven W. Singer[1,2], Héctor García Martín[1,2,5,6], Michelle A. O'Malley[1,4]

[1]Joint BioEnergy Institute, Emeryville, CA, USA

[2]Biological Systems and Engineering Division, Lawrence Berkeley National Laboratory, Berkeley, CA, USA

[3]Department of Chemical Engineering & Applied Chemistry, University of Toronto, Toronto, ON, Canada

[4]Department of Chemical Engineering, University of California, Santa Barbara, Santa Barbra, CA, USA

[5]DOE Agile BioFoundry, Emeryville, CA, USA

[6]BCAM, Basque Center for Applied Mathematics, Bilbao, Spain

[†]These authors contributed equally to this work

Supplementary Figures

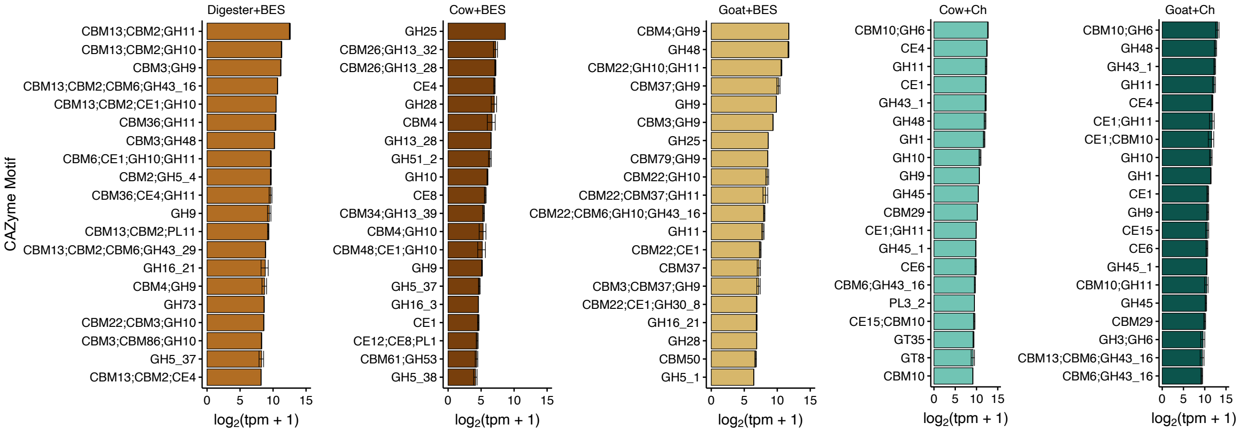

Supplementary Figure 1 – Top CAZyme motifs expressed across bacterial and fungal enrichments. CAZyme motifs were predicted using dbcanLight v1.0.2. BES – 2-bromoethansulfonate (bacterial enrichments), Ch-Chloramphenicol (fungal enrichments).

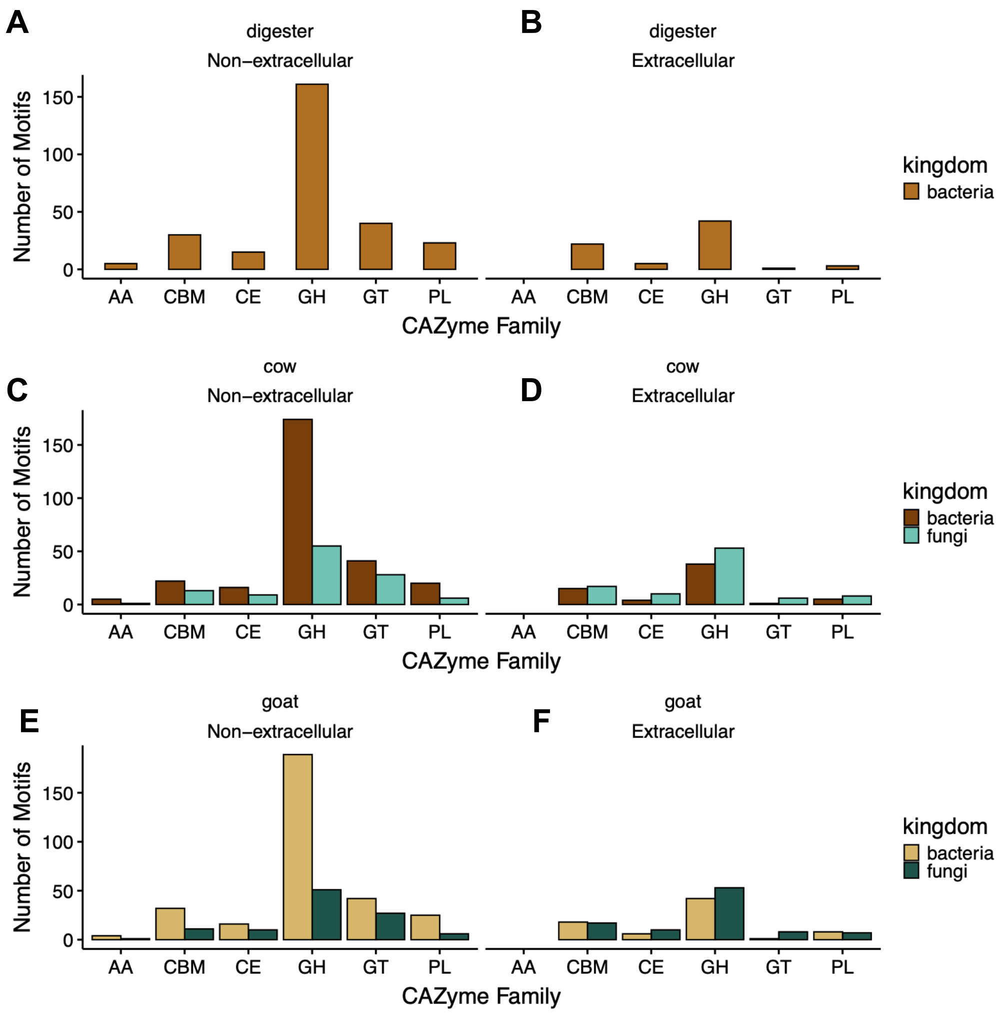

Supplementary Figure 2 - CAZyme family diversity by localization and enrichment source. Bar plots show the number of distinct CAZyme families – auxiliary activity (AA), carbohydrate-binding module (CBM), carbohydrate esterase (CE), glycoside hydrolase (GH), glycosyltransferase (GT), and polysaccharide lyase (PL) – expressed in digester (A, B), cow (C, D) and goat (E, F) enrichments. For each enrichment source, the count of distinct cazyme families is further partitioned into non-extracellular (A, C, E) and extracellular (B, D, F) fractions based on predicted subcellular localization using psotb or SignalP for bacterial or fungal enrichements, respectively. Bars are colored by the taxonomic kingdom of the enrichment. Bars represent the mean number of distinct CAZymes across n=2 samples.

Supplementary Datasets

Supplementary Data 1 – MAG Statistics. MAG statistics, including MAG size, GC content, completeness and redundancy, and DNA/mRNA read mapping, from cow (A), goat (B), and digester (C) bacterial enrichments.

Supplementary Data 2 – Bacterial MAG CAZyme expression table. Annotation and expression of CAZymes across all bacterial enrichment MAGs.

Supplementary Data 3 – Fungal CAZyme expression table. Annotation and expression of CAZymes across fungal reference genomes.

Supplementary Data 4 – MAG pathway summary. Predicted MetaCyc reactions and metabolic pathways across abundant MAGs recovered from cow, goat, and digester enrichments.
